# Infective Endocarditis Complicated by Acute Ischemic Stroke from Septic Embolus: Successful Solitaire FR Thrombectomy

**DOI:** 10.4021/cr235e

**Published:** 2012-11-20

**Authors:** Jackson J Liang, Kalkidan G Bishu, Nandan S Anavekar

**Affiliations:** a Department of Internal Medicine, Mayo Clinic, Rochester, USA; b Department of Cardiovascular Diseases, Mayo Clinic, Rochester, USA; c Department of Cardiovascular Diseases, Mayo Clinic, Rochester, USA

**Keywords:** Septic embolism, Endocarditis, Stroke, Mechanical thrombectomy, Solitaire

## Abstract

Infective endocarditis (IE) is often complicated by systemic embolization. Acute stroke due to septic emboli is a particularly dreaded complication. Optimal treatment for acute stroke in IE has not been well outlined. Fibrinolytic therapy may be associated with increased risk for hemorrhagic transformation in patients with acute stroke in the setting of IE. We present a case of IE complicated by acute stroke which was successfully treated with mechanical thrombectomy. This case illustrates a role of mechanical thrombectomy devices in this patient population.

## Introduction

Infective endocarditis (IE) is often complicated by systemic embolization. Acute stroke due to septic emboli is a particularly dreaded complication. Optimal treatment for acute stroke in IE has not been well outlined. Fibrinolytic therapy may be associated with increased risk for hemorrhagic transformation in patients with acute stroke in the setting of IE. We present a case of IE complicated by acute stroke which was successfully treated with mechanical thrombectomy. This case illustrates a role of mechanical thrombectomy devices in this patient population.

## Case Report

A 70-year-old woman was brought to the emergency department (ED) after being found down by her son in her home. She had a history of an old right posterior cerebral artery stroke which left her with mild left hemiparesis as well as a history of diabetes, coronary artery disease, and chronic atrial fibrillation for which she was anticoagulated on warfarin.

**Figure 1:**
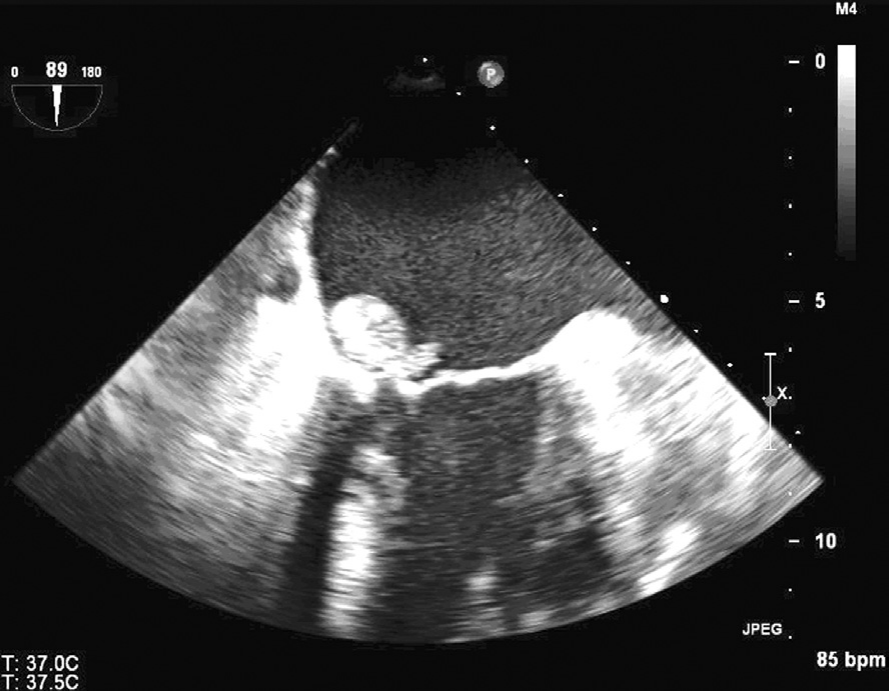
Transesophageal echocardiogram showing a 2 cm mobile mass on the atrial surface of the posterior mitral valve leaflet consistent with a vegetation.

On arrival to her local ED, her mental status was intact, but she was febrile to 39.5°Celsius and hypotensive with a blood pressure of 70/53. Heart rate was tachycardic at 120 beats per minute and tachypneic at 28 breaths per minute. She was admitted to the intensive care unit, where she was aggressively fluid resuscitated and started empirically on intravenous meropenem and vancomycin. Her blood pressure stabilized and she transferred to the floor, where multiple sets of blood cultures grew group B Streptococcus agalactiae. Transesophageal echocardiogram demonstrated a 2 cm mobile mass on the atrial surface of her posterior mitral valve leaflet (Fig. 1), consistent with vegetation due to IE. She was then transferred via ambulance to our tertiary hospital for management of IE.

**Figure 2:**
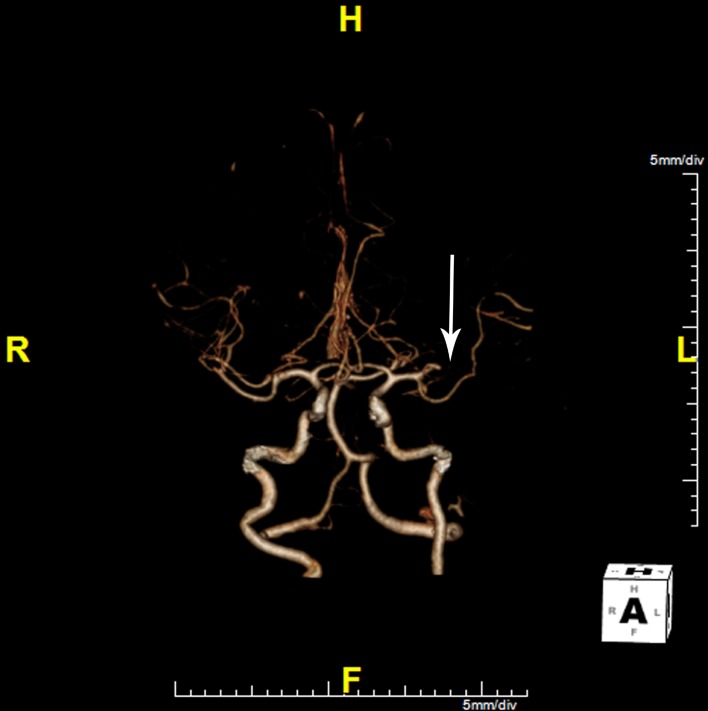
CT angiogram showing left MCA M2 occlusion (arrow).

**Figure 3:**
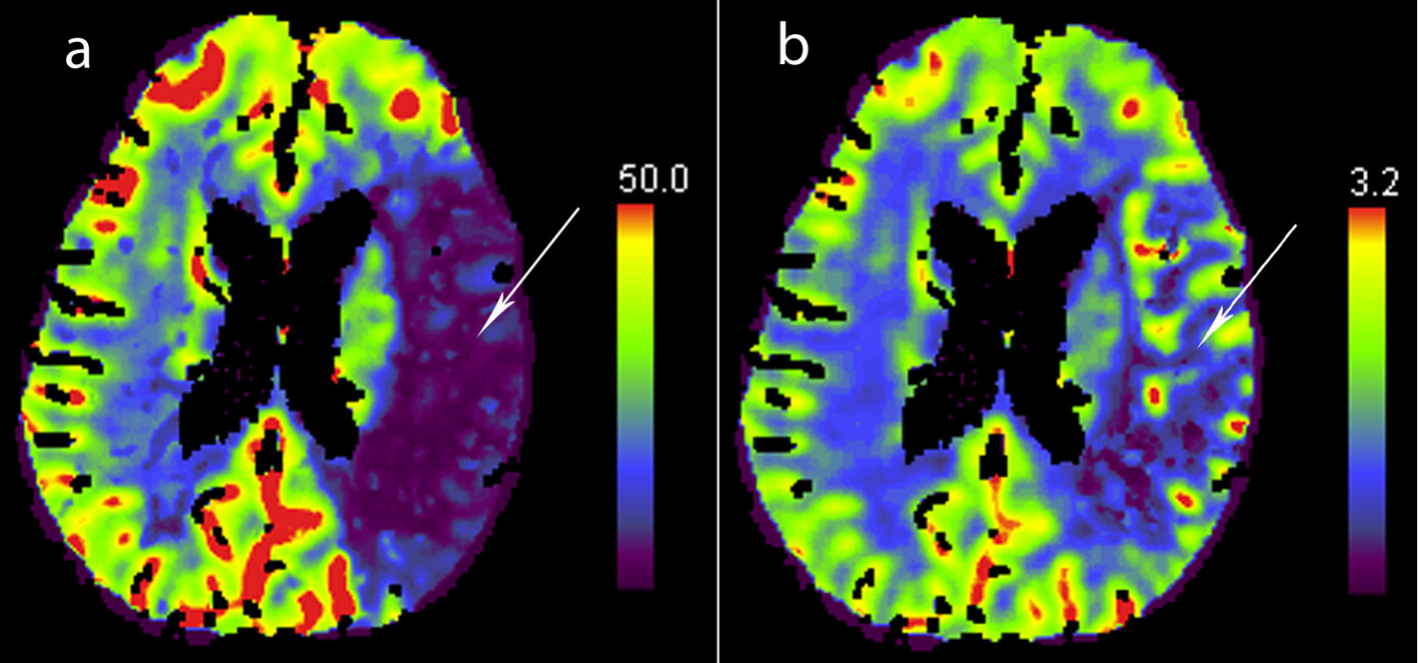
CT perfusion imaging demonstrating area of diminished CBF (a; arrow) with relatively normal CBV (b; arrow) suggesting evidence of salvageable penumbra.

En route to our hospital, she developed acute right-sided weakness and difficulty speaking. Upon arrival at our ED, she was noted to have both expressive and receptive aphasia with marked right hemiparesis. She was seen immediately by a neurologist and her NIH Stroke Scale score was determined to be 24. Physical exam demonstrated garbled speech, right hemiparesis, right sensory loss, right pronator drift, and a right Babinski sign. She became drowsy and developed respiratory distress, prompting intubation. Head CT without contrast revealed evidence of an old right occipital lobe infarct but no acute process. After hemorrhage was excluded, CT angiography (CTA) and CT perfusion (CTP) imaging were performed to evaluate for potentially salvageable ischemic penumbra or acute thrombus. CTA (Fig. 2) revealed complete abrupt occlusion of a large anterior left M2 segment. CTP (Fig. 3a, b) demonstrated an extensive area of diminished cerebral blood flow in the left middle cerebral artery (MCA) distribution, predominantly in the left frontal and parietal lobes. A moderate-sized penumbra of preserved cerebral blood volume was seen peripherally.

**Figure 4:**
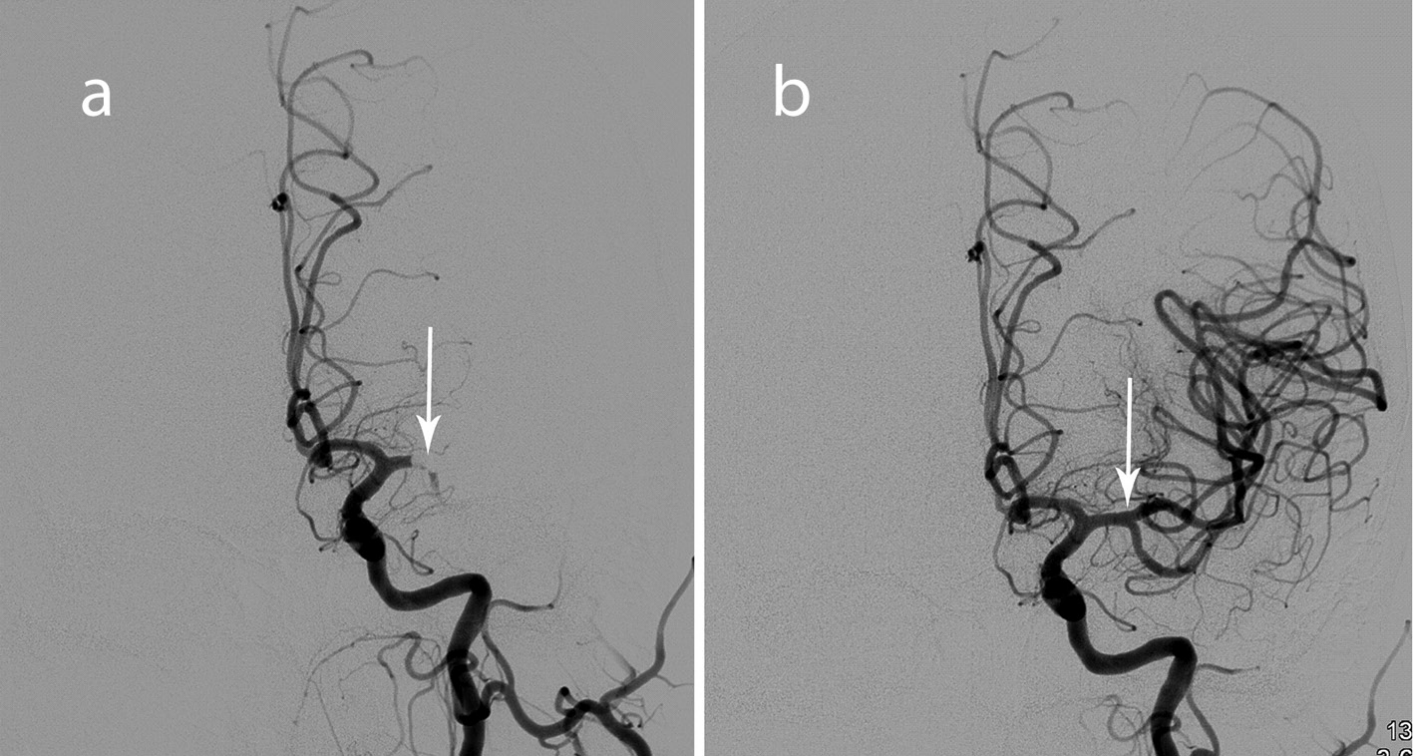
Angiogram images before (a) and after (b) mechanical embolectomy with restoration of flow through the left MCA M2.

Endovascular neuroradiology performed emergent intraarterial mechanical thrombectomy using the Solitaire FR revascularization device. Full recanalization was successfully achieved using the Solitaire retrievable stent (Fig. 4a, b). There were no procedural complications and post-procedure CT scan revealed no evidence of hemorrhage.

She experienced significant clinical improvement following the procedure. Her right-sided weakness began to recover within a few hours, and after one day she was able to move all extremities against gravity. By day 4, she exhibited no residual signs of her left MCA stroke.

She was deemed to be a poor surgical candidate due to her poor baseline functional status. Antibiotic regimen was deescalated to intravenous gentamicin and ceftriaxone. She was discharged to a skilled nursing facility to continue her rehabilitation and complete her course of antibiotics.

## Discussion

To our knowledge, this is only the second case report describing the successful retrieval of a septic embolus in the setting of IE using the Solitaire FR device. The first report described a 33-year-old man with IE and septic embolism causing an acute M1 occlusion which was also successfully revascularized [1]. There has also been one case of successful retrieval of a septic embolus using the Penumbra aspiration system described in the literature [2].

Acute ischemic stroke secondary to septic emboli is a dreaded complication of IE. Stroke is the initial presenting sign in 4-14% of patients with IE [3]. Embolization occurs more frequently with larger ( > 10mm), left-sided vegetations, particularly on the mitral valve [4], and when antiphospholipid antibodies are present [5]. Although IV tPA improves clinical outcomes in acute ischemic stroke if given within 4.5 hours after onset of symptoms [6], it may increase risk for hemorrhagic conversion in patients with strokes related to IE [7]. Furthermore, vegetations comprise inflammatory cells, platelets and microorganisms in addition to a rich fibrin network [8]. As such, IV tPA may not be as effective in the setting of septic emboli.

The most common cause of intracranial hemorrhage in patients with IE related to septic emboli is hemorrhagic transformation of the ischemic infarct [9] and administration of tPA potentiates this risk. Mechanical thrombectomy is an option for reperfusion of intracranial vessels within 8 hours of symptom onset, allowing for reperfusion without the risk of hemorrhage that IV and intra arterial fibrinolytics carry. There are multiple mechanical thrombectomy devices currently in use such as the Merci, Phenox, and Catch clot retrieval devices and the Penumbra system, which is a vacuum device. The Solitaire FR device is an intracranial stent that is deployed in an occluded vessel and allows for withdrawal of the thrombus once unfolded [10]. Successful revascularization occurs in upwards of 90% of patients treated with this device [11].

Prevention of systemic embolization remains an important aspect in the treatment of IE. Immediate initiation of appropriate antibiotics decreases risk of septic embolization to the brain [12]. Prior daily antiplatelet and statin [13] therapy before diagnosis of IE may prevent septic embolization. Contrarily, initiation of aspirin after diagnosis of IE has no benefit and may increase risk of bleeding [14].

## Conclusion

Mechanical thrombectomy is a treatment option for acute stroke due to septic embolus from IE if performed within 8 hours of symptom onset. Rapid transfer to an institution with the capability to perform such procedures can be a consideration in these patients, although long term experience in this setting is still lacking.
